# Gestational weight gain and estimated fetal growth in different trimesters among Saudi women

**DOI:** 10.12669/pjms.41.11.11278

**Published:** 2025-11

**Authors:** Iffat I Nazir

**Affiliations:** Dr. Iffat I Nazir, FCPS-Obstetrics & Gynecology, MHPE Assistant Professor, Obstetrics & Gynecology Department, College of Medicine, Taif University, Saudi Arabia

**Keywords:** Gestational weight gain, Fetal weight, Gestational age, Birth weight

## Abstract

**Objectives:**

Maternal weight gain during pregnancy affects fetal growth. This study aimed to determine the association between gestational weight gain (GWG) and fetal weight gain at different weeks of gestation. The study also intended to determine the relationships among maternal pre-pregnancy body mass index (BMI), GWG, and neonatal birth weight.

**Method:**

This prospective observational cohort study examined 316 pregnant women at Security Forces Hospital, Makkah, from July 1st, 2023, to June 30th, 2024. All uncomplicated, singleton, booked cases with term deliveries were included. Maternal pre-pregnancy weight and BMI were determined and categorized as normal, overweight, or obese. Participants were followed up with from 18 ± 1 weeks until delivery. Gestational weight gain and estimated fetal weight by ultrasound were recorded and compared at weeks 18, 24, 28, 32, and 36.

**Results:**

GWG showed no significant correlation with estimated fetal weight at 18 weeks (B=0.001, p=0.242) and 24 weeks (B=0.003, p=0.242). However, GWG at 28 weeks (B=0.044, p<0.001), 32 weeks (B=0.029, p=0.001), and 36 weeks (B=115.13, p<0.001) strongly predicted fetal weight at the corresponding weeks. Pre-pregnancy weight (r=0.10, p=0.073) and BMI (r=0.08, p=0.164) showed non-significant correlations with neonatal birth weight. However, total GWG (B=0.068, p<0.001) and pregnancy duration (B=20.81, p<0.001) significantly influenced birth weight. Despite variations in GWG, average birth weights were relatively similar across all BMI maternal groups, with the obese group having the highest birth weights, followed by the overweight and normal groups.

**Conclusion:**

Maternal GWG directly affects fetal weight (especially during the third trimester) and birth weight.

## INTRODUCTION

Intrauterine fetal growth relies on hereditary, maternal, fetal, and placental factors.^1^ Acceptable birth weight ranges from 2500 to 4000 g (10th–90th percentile); macrosomia is above 4000 g, and low birth weight (LBW) is below 2500 g.^2^ Birth weight indicates intrauterine growth, correlates with obstetric complications, and affects long-term survival.^3^ Pre-pregnancy weight, body mass index (BMI), and gestational weight gain (GWG) are key modifiable maternal factors.^4^ Insufficient GWG can cause preterm delivery, LBW, and perinatal mortality, while excessive GWG can lead to macrosomia, gestational diabetes, birth trauma, and operative deliveries.^5^ Maternal weight is monitored during prenatal visits as an indirect measure of fetal growth.^6^ Prenatal ultrasound directly estimates fetal growth by measuring various parameters.^7^

A meta-analysis of 45 studies found that women with low pre-pregnancy BMI were more likely to deliver small-for-gestational-age and LBW babies. Conversely, women with high pre-pregnancy BMI are more prone to having large-for-gestational-age and macrosomic infants.^8^ Another systematic review of 35 studies noted that uncontrolled weight gain during pregnancy is associated with large-for-gestational-age infants. In contrast, insufficient weight gain is linked to LBW and small-for-gestational-age infants.^9^

GWG significantly influences fetal growth and birth weight, but its temporal distribution during pregnancy may have critical implications. Limited research exists on weight gain patterns in mothers and fetuses at different pregnancy stages. This study aimed to evaluate maternal weight gain patterns during various pregnancy periods based on ultrasound-estimated fetal weight and to observe the effects of pre-pregnancy BMI and GWG on neonatal birth weight.

## METHOD

This prospective observational cohort study was performed at Security Forces Hospital, Makkah, Saudi Arabia. The study spanned from July 1, 2023, to June 30, 2024. It included all willing, booked, singleton pregnant mothers. Participants were explained the study objectives and informed consent was taken. The study excluded pregnancies with complications, uncertain due dates, fetal malformations, and delivering pre-term or post-term. A sample size of 285 was calculated using a 95% confidence level and 90% power, with an expected correlation of 0.19 between birth weight and pre-pregnancy weight.^10^

### Ethical Approval:

Ethical approval was obtained by SFHM IRB with IRB# 0491-030722 on May 4^th^, 2023.

A non-probability convenience sampling technique was used. Eligible expectant mothers were monitored from 18 ± 1 weeks until delivery, yielding 316 complete cases. Maternal details including age, height, pre-pregnancy weight and BMI, obstetric history, and employment status were noted. Pre-pregnancy weight was recorded at the initial booking visit at 6–8 weeks or via self-reporting. Pre-pregnancy BMI was categorized as underweight (18.5–24.9 kg/m²), normal (20–24.9 kg/m²), overweight (25–29.9 kg/m²), and obese (30–>40 kg/m²).

Gestational weight gain (GWG) and estimated fetal weight (EFW) were measured at five intervals: 18 ± 1 weeks (GWG1-18, EFW1-18), 24 ± 1 weeks (GWG2-24, EFW2-24), 28 ± 1 weeks (GWG3-28, EFW3-28), 32 ± 1 weeks (GWG4-32, EFW4-32), and 36 ± 1 weeks (GWG5-36, EFW5-36). Maternal weight at birth was recorded at delivery admission or the last antenatal visit within two weeks of delivery. EFW was determined using ultrasonography.

### Statistical Analysis:

Data was analyzed using SPSS version 27. GWG was compared with EFW at each interval to assess maternal-fetal weight gain patterns. Total GWG was categorized based on pre-pregnancy BMI. The U.S. Institute of Medicine’s 2009 guidelines recommended total GWGs for underweight, normal-weight, overweight, and obese women as 12.5–18 kg, 5–16 kg, 7–11.5 kg, and 5–9 kg, respectively.^11^ Neonatal birth weight was classified as low (below 2.5 kg), normal (2.5–4 kg), and macrosomic (over 4 kg).

Descriptive data were presented as frequencies and percentages or mean values and standard deviations (SD). Pearson’s correlation coefficient assessed the association between GWG and EFW. Multiple linear regression analyses examined how independent variables predicted EFW at 18, 24, 28, 32, and 36 weeks and birth weight.

## RESULTS

This study included 316 pregnant women aged 18 to 42 years with a mean age of 30 years. The majority of participants were housewives (67.7%), 16.8% were employed, and a small percentage were students. Regarding gravidity, 25.9% of the participants were primigravida, 64.9% were multigravida, and 9.2% were grand multigravida. Pre-pregnancy BMIs ranged from 18.8 to 41.4 kg/m², with a mean of 26.7 kg/m² (SD ± 5.1). The BMI distribution was 45.3% with normal BMI, 32.3% being overweight, and 22.4% obese. The total GWG revealed 14.2% below normal, 38.0% normal, and 47.8% excessive weight gain. The mean gestation period was 38.8 weeks (SD ± 1.0), with the majority of deliveries occurring between 38.2 and 39.5 weeks; 17.7% of deliveries were before 38 weeks, 37.7% between 38.0-38.9 weeks, 22.2% between 39.0-39.9 weeks, and 22.5% after 40 weeks. Regarding delivery methods, 60.4% were normal vaginal deliveries (NVD), 22.5% were elective cesarean sections (CS), and 17.1% were emergency CS. Neonate gender distribution indicated 57.3% female and 42.7% male newborns. Birth weight classification revealed 7.0% low birth weight (LBW), 87.0% normal weight, and 6.0% macrosomic. Table-I.

**Table-I:** Gestational weight gain at each successive gestational interval (in weeks) and neonatal characteristics.

Variables	Mean	SD	Median	Q1	Q3	Minimum	Maximum
GWG1-18 (gm)	2375	899	2400	1700	3000	600	5200
GWG2-24 (gm)	4452	1193	4500	3500	5200	2000	8000
GWG3-28 (gm)	6581	1405	6500	5700	7500	3500	12000
GWG4-32(gm)	8796	1723	8500	7500	9500	5000	14500
GWG5-36 (gm)	10989	1944	10500	9500	12200	6400	16200
Total GWG (gm)	12660	1996	12550	11200	14050	7100	18000
EFW5-36 (gm)	2815	321	2813	2609	3146	2151	3624
Birth weight (gm)	3145	440	3070	2860	3408	2330	4260
Baby length (cm)	50	2	49	48	50	47	55
Baby HC* (cm)	34	1	34	34	35	31	36
Difference in birth weight as compared to EFW5- 36 weeks	329.79	205.08	281.50	179.00	450.00	-60.00	1119.00

* Head circumference

Gestational weight gain (GWG) measured at 18, 24, 28, 32, and 36 weeks showed positive and moderately strong correlations with subsequent GWG values (Table-II). Also, the correlations between GWG and EFW and GWG were generally modest but became stronger as the pregnancy progressed, with many values reaching statistical significance in later weeks. For example, at 18 weeks GWG1 showed a correlation of 0.10 with EFW1, which increased slightly by 36 weeks (GWG5 with EFW5, r = 0.23, p < 0.001).

**Table-II:** Correlations between Gestational Weight Gain (GWG) and Estimated Fetal Weight (EFW) across pre-defined pregnancy weeks.

Varia bles	GWG1 18	GWG2 24	GWG3 28	GWG4 32	GWG5 36	EFW1 18	EFW2 24	EFW3 28	EFW4 32	EFW5 36
GWG1 18	1.00	0.78	0.69	0.59	0.53	0.10 (0.075)	0.08	0.13 (0.026)	0.12 (0.030)	0.20 (<0.001)
GWG2 24	0.78	1.00	0.86	0.74	0.69	0.06	0.06 (0.299)	0.15 (0.008)	0.13 (0.020)	0.17 (0.003)
GWG3 28	0.69	0.86	1.00	0.87	0.81	0.08	-0.04	0.25 (<0.001)	0.15 (0.010)	0.18 (0.001)
GWG4 32	0.59	0.74	0.87	1.00	0.93	0.14	0.04	0.23	0.19 (0.001)	0.22 (<0.001)
GWG5 36	0.53	0.69	0.81	0.93	1.00	0.11	0.03	0.20	0.19	0.23 (<0.001)
EFW1 18	0.10	0.06	0.08	0.14	0.11	1.00	0.23 (<0.001)	0.02	0.02	0.07
EFW2 24	0.08	0.06	-0.04	0.04	0.03	0.23	1.00	0.07	0.18 (0.001)	0.12 (0.032)
EFW3 28	0.12	0.15	0.25	0.23	0.20	0.02	0.07	1.00	0.11 (0.041)	0.17 (0.003)
EFW4 32	0.12	0.13	0.15	0.19	0.19	0.02	0.18	0.11	1.00	0.35 (<0.001)
EFW5 36	0.20	0.17	0.18	0.22	0.23	0.07	0.12	0.17	0.35	1.00

Regression analysis identified key factors affecting EFW throughout pregnancy and birth weight (Table-III.) At 18 weeks, the model accounted for 14.5% of the variance in EFW, with pre-pregnancy weight showing a significant positive effect (B=4.05, p<0.001) and BMI demonstrating a negative effect (B=-9.53, p=0.001). GWG 1-18 had no significant impact (B=0.001, p=0.242). Strong predictors of fetal weight were GWG3 at 28 weeks (B=0.044, p<0.001), GWG4 at 32 weeks (B=0.029, p=0.001), and GWG5 at 36 weeks (B=115.13, p<0.001). Total gestational weight gain (B=0.068, p<0.001) and gestational age at delivery (B=20.81, p<0.001) were the determinants of birth weight.

**Table-III:** Regression Analysis of Maternal and Gestational Factors Influencing Estimated Fetal Weight and Birth Weight.

	R^2^ = 0.145		R^2^ = 0.048		R^2^ = 0.068		R^2^ = 0.076		R^2^ = 0.164		R^2^ = 0.289	
Variable	B	P-value	B	P-value	B	P-value	B	P-value	B	P-value	B	P-value
Constant	740.2	<0.001	157.9	0.827	-2091.4	0.285	-1611.1	0.481	-4035.3	0.118	-7699.8	0.018
Age	0.03	0.895	0.49	0.560	2.16	0.340	-1.41	0.593	-3.63	0.224	-4.69	0.214
Gravidity	3.75	0.061	6.27	0.472	-20.39	0.388	30.80	0.267	112.31	<0.001	115.13	0.004
Height	-2.75	0.005	4.14	0.326	12.19	0.285	10.23	0.442	15.57	0.301	22.24	0.243
BMI	-9.53	0.001	14.18	0.267	27.18	0.432	16.18	0.688	34.75	0.446	40.49	0.483
Mat Weight	4.05	<0.001	-5.27	0.291	-10.61	0.433	-5.06	0.748	-13.33	0.455	-14.89	0.509
GA at delivery	-0.39	0.011	-1.12	0.098	2.16	0.237	4.06	0.058	11.97	<0.001	20.81	<0.001
Gender (male baby)	-4.51	0.045	12.14	0.217	4.41	0.868	35.61	0.252	59.29	0.092	96.39	0.030
Mode of delivery	1.81	0.213	11.31	0.078	15.57	0.369	15.75	0.436	31.49	0.169	85.88	0.003
Baby length	0.32	0.607	0.99	0.715	6.54	0.377	9.60	0.269	8.75	0.371	6.82	0.580
GWG1-18	.001	0.242	--	--	--	--	--	--	--	--	--	--
GWG2-24	--	--	0.003	0.476	--	--	--	--	--	--	--	--
GWG3-28	--	--	--	--	0.044	<0.001	--	--	--	--	--	--
GWG4-32	--	--	--	--	--	--	0.029	0.001	--	--	--	--
GWG5-36	--	--	--	--	--	--	--	--	0.037	<0.001	--	--
Total GWG	--	--	--	--	--	--	--	--	--	--	0.068	<0.001
Dependent Variable	EFW1-18		EFW2-24		EFW3-28		EFW4-32		EFW5-36		Birth weight	

Bold face coefficients are all significant at 0.05 level of significance; Grey-shaded coefficients are considerable as p-values are ≤0.100

Certain other correlations included a weak correlation between pre-pregnancy weight and neonatal birth weight (r = 0.10, p = 0.073), and an even weaker correlation between BMI and neonatal birth weight (r = 0.08, p = 0.164). However, GWG showed a moderate, significant positive correlation with neonatal birth weight (r = 0.36, p < 0.001), indicating that GWG is more closely related to birth weight than to pre-pregnancy weight or BMI. Additionally, pre-pregnancy weight and BMI had a strong correlation (r = 0.94) and weak but significant correlations with total GWG (r = 0.20 and r = 0.21, respectively; p < 0.001), suggesting a strong correlation with GWG but a less prominent effect on birth weight.

## DISCUSSION

The study reveals a positive association between GWG and fetal weight gain across all gestational periods. Notably, maternal weight gain correlations are stronger at 28, 32, and 36 weeks (p < 0.001, 0.001, and < 0.001, respectively) compared to 18 and 24 weeks (p = 0.242 and 0.476, respectively). This suggests that early pregnancy weight gain provides some insight into fetal weight, but its predictive power improves significantly in later pregnancy stages. This finding concurs with Milosevic and Kozarov’s study, which found a third-trimester correlation between maternal weight gain and estimated fetal weight, but none in the first or second trimesters.^12^ Conversely, Retnakaran et al. reported that only weight gain from pre-pregnancy to the early second trimester correlated significantly with infant birth weight, whereas later weight gain did not.^13^

Similarly, Lin et al. in a Chinese study, identified 14 to 23 weeks of pregnancy as the most critical period affecting fetal weight.^14^ Pregnancy involves a complex endocrine-metabolic rearrangement, where maternal weight gain supports optimal fetal development. Changes in maternal body composition aid fetal growth, with early pregnancy marked by changes in the uterus, breast tissue, and blood volume. Initially, the fetus does not accumulate fat or muscle, but significant growth occurs towards the end of pregnancy.^15^ Maternal insulin resistance ensures essential nutrients reach the fetus. Maternal weight gain increases insulin resistance and alters hormones regulating placental nutrient transporters, leading to increased fetal insulin release, growth, and weight gain in the last trimester.^16^ Regarding the GWG, a mean of 12.7 (SD ± 2.0) kg GWG was observed, which corroborates other studies.^17^,^18^ The study shows a clear separation of GWG patterns between the obese group and the other two groups, particularly after 24 weeks, when the obese group diverged significantly and gained more weight on average.

The normal and overweight groups followed a similar trend, with the overweight group consistently gaining slightly more weight than the normal group did. Despite differences in gestational weight gain, the average birth weights across the groups were relatively similar, with the obese group having the highest average birth weight, followed by the overweight and normal groups. Similar observations were reported by Nowak et al. in a Polish study.^19^ The overall findings of the current study revealed that normal GWG is generally associated with favorable birth weight outcomes. Interestingly, normal GWG was significantly associated with increased birth weight risk in mothers with overweight BMIs, and below-normal GWG was more likely to be associated with LBW in mothers with BMIs classified as normal. Table-IV A comparable pattern regarding gestational weight gain (GWG) and infant birth weight among overweight and obese mothers was observed in a recent study conducted in Thailand.^20^

**Table-IV:** Distribution of Birth Weight Across Total Gestational Weight Gain Levels and BMI Categories.

Total GWG levels	Birth weight	Maternal pre-pregnancy BMI category			Total	P-value
			Normal	Overweight	Obese		
Below normal	LBW*	Count	10	0		10	0.561
		%	24.4	0.0		22.2	
	Normal weight	Count	31	4		35	
		%	75.6	100.0		77.8	
	Total	Count	41	4		45	
		%	100.0	100.0		100.0	
**Normal**	LBW*	Count	0	5	0	5	0.012
		%	0.0	13.5	0.0	4.2	
	Normal weight	Count	78	30	2	110	
		%	96.3	81.1	100.0	91.7	
	Macrosomia	Count	3	2	0	5	
		%	3.7	5.4	0.0	4.2	
	Total	Count	81	37	2	120	
		%	100.0	100.0	100.0	100.0	
**Excessive**	LBW*	Count	0	5	2	7	0.244
		%	0.0	6.1	3.2	4.6	
	Normal weight	Count	5	68	57	130	
		%	71.4	82.9	91.9	86.1	
	Macrosomia	Count	2	9	3	14	
		%	28.6	11.0	4.8	9.3	
	Total	Count	7	82	62	151	
		%	100.0	100.0	100.0	100.0	
**Total**	LBW*	Count	10	10	2	22	0.253
		%	7.8	8.1	3.1	7.0	
	Normal weight	Count	114	102	59	275	
		%	88.4	82.9	92.2	87.0	
	Macrosomia	Count	5	11	3	19	
		%	3.9	8.9	4.7	6.0	
	Total	Count	129	123	64	316	
		%	100.0	100.0	100.0	100.0	

* Low birth weight

Although GWG was found to have a strong positive correlation with fetal weight and neonatal birth weight, no such correlation was observed in this study for maternal pre-pregnancy weight or BMI and birth weight. Chen et al. and Mohammadnuri et al. found that maternal BMI positively influences fetal growth and birth weight.^21^,^22^ Interestingly, BMI was significantly associated with maternal GWG in the current study and GWG was strongly associated with neonatal birth weight. This finding implies an indirect effect of pre-pregnancy BMI on neonatal birth weight. Very similar findings were documented by Lackovic et al.^23^ These varying observations can be attributed to the diverse factors influencing fetal growth. Weight gain may be associated with excessive birth weight due to shared obesity-related genes between the mother and infant.^24^

### Strengths

The strengths of this study are its simultaneous maternal and fetal weight measurement protocols at five predefined pregnancy intervals and prospective data collection.

### Limitations

This study offers valuable insights but has certain limitations, including self-reported pre-pregnancy weight and only two participants in the underweight BMI category. The sample may not represent all demographics, potentially affecting the generalizability of the results.

Future research should involve larger, more diverse cohorts and adopt longitudinal designs to better understand the interactions between maternal weight status, gestational weight gain (GWG), and fetal development throughout pregnancy. Additionally, tracking pregnant women from the onset of pregnancy is recommended.

## CONCLUSIONS

Gestational weight gain significantly predicted estimated fetal and birth weights, particularly in the latter half of the pregnancy. Pre-pregnancy weight and BMI initially influenced fetal weight but subsequently slowed down. Variations in birth weight were related to gestational age at delivery, mode of delivery, and the male sex of the baby. This study underscores the importance of monitoring maternal weight gain during pregnancy because of its impact on fetal development and birth outcomes.
